# Single anthocyanins effectiveness modulating inflammation markers in obesity: dosage and matrix composition analysis

**DOI:** 10.3389/fnut.2023.1255518

**Published:** 2023-11-02

**Authors:** Jorge Alberto Fragoso-Medina, Selma Romina López Vaquera, Astrid Domínguez-Uscanga, Diego Luna-Vital, Noemí García

**Affiliations:** ^1^Institute for Obesity Research, Tecnologico de Monterrey, Monterrey, Mexico; ^2^School of Medicine and Health Sciences, Tecnologico de Monterrey, Monterrey, Mexico; ^3^School of Engineering and Sciences, Tecnologico de Monterrey, Monterrey, Mexico; ^4^Preclinical Research Unit, Tecnologico de Monterrey, Monterrey, Mexico

**Keywords:** antioxidants, anti-inflammatories, anthocyanins bioactivities, anthocyanins composition, single anthocyanins doses, inflammation markers

## Abstract

Anthocyanins (ACNs) are phytochemicals with numerous bioactivities, e.g., antioxidant and anti-inflammatory. Health benefits from consuming ACN-rich foods, extracts, and supplements have been studied in clinical trials (CT). However, the individual effect of single ACNs and their correlation with doses and specific bioactivities or molecular targets have not been thoroughly analyzed. This review shows a recompilation of single anthocyanins composition and concentrations used in CT, conducted to investigate the effect of these anti-inflammatory derivatives in obese condition. Single anthocyanin doses with changes in the levels of frequently monitored markers were correlated. In addition, the analysis was complemented with reports of studies made *in vitro* with single ACNs. Anthocyanins' efficacy in diseases with high baseline obesity-related inflammation markers was evidenced. A poor correlation was found between most single anthocyanin doses and level changes of commonly monitored markers. Correlations between cyanidin, delphinidin, and pelargonidin derivatives and specific molecular targets were proposed. Our analysis showed that knowledge of specific compositions and anthocyanin concentrations determined in future studies would provide more information about mechanisms of action.

## 1. Introduction

**Figure 1:**
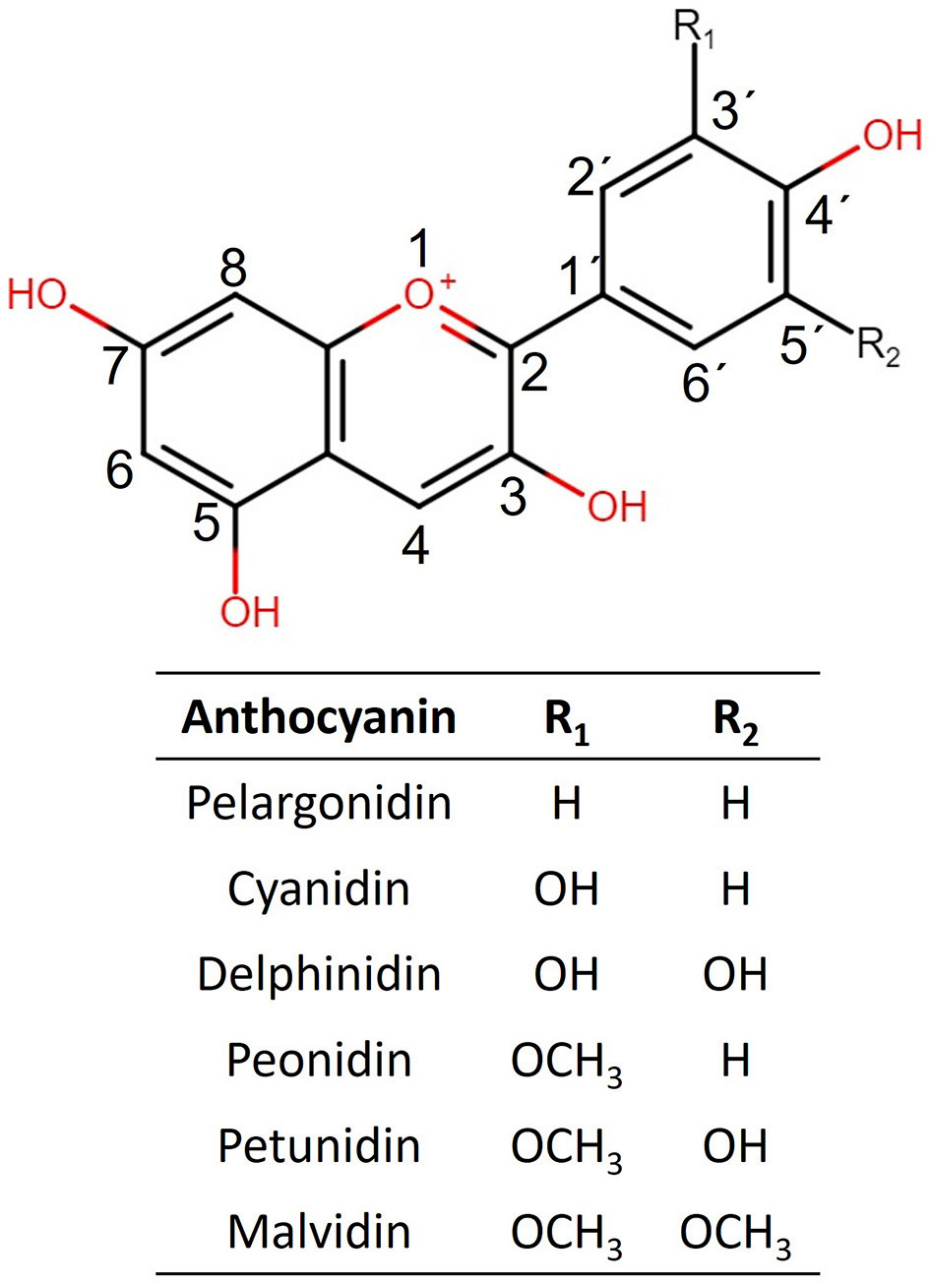
Structures of the six more commonly naturally occurring anthocyanidins are shown.

Anthocyanins (ACNs) are a group of phenolic compounds belonging to flavonoids, including other compounds such as flavones, flavanols, flavonols, flavanones, and isoflavones. ACNs are secondary metabolites that confer characteristic color to many plants such as black soybean seed, purple potato, purple cabbage, black carrot, purple corn, and a great variety of berries (1, 2). Their characteristic brilliant colors have propitiated their use as pigments; therefore, the commercial interest in natural sources producing them has increased. Chemically, ACN color depends on the pH environment, ranging from blue in alkaline conditions to purplish red in acid ones. Their structure comprises an anthocyanidin substituted with a carbohydrate in the 3-O' position, although the 5-O' substituted anthocyanidins are also common (Figure 1). As ACN-rich foods have been associated with antioxidant, anti-inflammatory, and anti-obesogenic activities (3), their adjuvant and therapeutic potential has been widely assessed in the last decade.

Due to the common sources of many flavonoids, several bioactivities previously associated with flavonoids have been recently attributed to ACNs, e.g., antioxidant, anti-inflammatory, hepatoprotective, and vasorelaxant activities are shared by ACN-free flavonoids and ACNs. Additionally, many of the molecular targets, namely cytokines, free radicals, and signal transduction proteins associated with flavonoids, have also been related to ACNs (2–6). This fact could be a result of their common molecular structures (4). For example, the absence of a carbonyl double bond in C3 in ACNs would modify potency in many biological activities when purified ACN extracts were used. Regarding differences among ACN extracts, standardized bilberry extracts have been widely used as supplements, and the non-ACN fraction from these extracts has been suggested to improve the efficacy of vision disorder treatments, presumably due to the carbohydrate content not preserved in several extracts available on the market (7). The carbohydrate content or other components in ACN-rich sources may improve, modify, or reduce ACN efficacy. However, not enough studies have been conducted to understand this controversy. This fact raises the question, what are the specific compositions of ACN-rich foods and extracts used in research conducted in this field?

Numerous review papers have proposed using anthocyanins to treat inflammation-related diseases (2, 3, 5). In this respect, a systematic review of components in five diets rich in polyphenols used in clinical trials (CTs) found that weekly polyphenol consumption could vary up to three-fold (8). However, because this study was designed to propose optimal polyphenol-rich diets, there is little information about the effects of single compounds and their absorption.

### 1.1. Anthocyanin bioavailability mechanism

For any bioactive compound to exert its effect, bioavailability represents a key factor and depends mainly on its structure, which determines its absorption, distribution, metabolism, and excretion. Additionally, when incorporated as ingredients, other aspects influencing bioavailability are the quality of the food matrix, food processing, and interaction with other food matrices. Despite all those factors, important mechanisms and aspects of ACN metabolism have been established that could help to explain whether specific ACNs are preferentially absorbed. The first step of ACN metabolism occurs in the oral cavity, where ACNs are partially degraded by a glucosidase enzyme (9). Once ACNs reach the stomach, 20–25% can be rapidly absorbed, crossing the gastric mucosa with their intact form. In gastric mucosa, ACNs are internalized by membrane transporters such as SMCT1, SMCT2, GLUT1, OAT2, or bilitranslocase-mediated absorption (10). After reaching the small intestine, ACN stability is greatly reduced due to the neutral-to-mildly alkaline environment (pH 7.5–8) and converted to hemiketal or chalcones (11).

Regarding absorption of anthocyanin glycosides, it has been reported that the highest rate occurs in the jejunum and ileum; however, there still can be a small rate of absorption in the duodenum (11, 12). In the small intestine, there are two potential mechanisms of ACN absorption: one involving the glucose transporters SGLT1 and GLUT2, and another possible mechanism is the hydrolysis of ACNs by brush border enzymes such as lactase phloridzin hydrolase before passive diffusion of the aglycone (13, 14). The remaining unabsorbed ACNs are metabolized by intestinal microbiota. ACNs serve as the substrate for enzymes located in the small intestine, colon, and liver, particularly hydrolyzing, and phase I (cytochrome P450 monooxygenase system and Flavin-containing monooxygenase system) and phase II (uridine-5'-diphospho-glucuronosyltransferase, sulfotransferase, and catechol-O-methyltransferase) enzymes (15).

On the other hand, the hydrolysis of anthocyanins is mainly caused by the β-D-glucosidase, β-D-glucuronidase, α-galactosidase, and α-rhamnosidase activity of gut microbiota to aglycones and small phenolic acids (16, 17). Then, phase II enzymes convert the resulting catabolites to glucuronides, methylates, and sulfates in the liver and kidneys. Conjugated forms of anthocyanins and their metabolites may be excreted via bile to the jejunum and recycled by the enterohepatic circulation system (15).

Summarizing current knowledge about using ACNs against inflammation, efforts have been made to describe some possible involved mechanisms, polyphenol composition of diets, and how they are metabolized. However, there is still a need to understand how concentrations and identities of single ACNs are related to their effects since different molecules might act synergistically or antagonistically and mask potential intensified efficacies. Particularly, inflammation markers are obesity targets (3, 5) that might help to draw a relationship between specific activities and single ACNs. Therefore, in this review, obesity-related inflammation reports were statistically analyzed to determine if currently, available reports could reflect specific effects of single ACNs (ACNs isolated individually) that could improve health.

## 2. Methods

### 2.1. Literature review for absorption and pharmacokinetics of anthocyanins-rich matrices

Studies investigating the identity of single ACNs and metabolites absorbed from different sources in the last 10 years (2012–2022) were reviewed. The main pharmacokinetic parameters are summarized in Table 1.

**Table 1:** Evaluation of absorption and pharmacokinetics of anthocyanins-rich matrices.

**Source**	**ACN/metabolites found**	**Pharmacokinetics parameters**		**References**
		**Plasma**	**Urine**	
Red raspberries	Sixteen phenolic metabolites derived from ACNs detected in urine. Nine ACN metabolites were found in plasma. Cyanidin-O-glucuronide found at 2 h, 4 h, and 6 h, at concentrations that ranged from 0.1 to 0.3 nmol/L	Cyanidin-3-O-glucoside t_max_= 1.0 h, C_max_= 0.2 nM	Cyanidin-3-O-glucoside t_max_= 0–4 h, Max. excretion = 9.3 nmol, Excreted % (48 h): 0.054 Peonidin-3-O-glucoside t_max_= 0–4 h, Max. excretion = 0.9 nmol^a^	(18)
Table red wine/young Port red wine	Pn3glc; Mv3glc; DpGlucr; PnGlucr; MvGlucr found in plasma and urine after table red wine consumption. Mv3glc; PnGlucr; MvGlucr were found in plasma after young port consumption. Pn3glc and DpGlucr were not found in urine after young port consumption	Table red wine total ACNs t_max_= 120 min, C_max_= 32.29 mg ml ^−1^ of total ACNs and metabolites Young port red wine t_max_= 90 min, C_max_= 5.90 mg ml ^−1^ of total ACNs and metabolites	NA	(19)
Commercially available wild blueberry juice	MS/MS search included 18 parent ACNs and 41 predicted ACN metabolites	NA	Anthocyanins and derivatives t_max_= 28 days C_max_ total aglycones = 5,492 nmol, Excreted % (24 h): 1.226 C_max_ aglycone glucuronides = 4,181 nmol, Excreted % (24 h): 0.933 C_max_ simple aglycones = 1,310 nmol, Excreted % (24 h): 0.292 C_max_ total glycosides = 423 nmol, Excreted % (24 h): 0.094 Parent ACNs = 240.7 nmol, Excreted % (24 h): 0.054	(20)
Red grape pomace	Ten hydroxybenzoic acids and simple phenols were detected in plasma, being methylpyrogallol-sulfate and proto-catechuic acid-3-sulfate the most representative compounds. Nine simple phenols and hydroxybenzoic acids were detected in urine	Methylpyrogallol-sulfate t_max_= 3.6 h, C_max_= 512.4 nM Protocatechuic acid-3-sulfate t_max_= 3.8 h C_max_= 408.5 nM	Total simple phenols and hydroxybenzoic acids t_max_= 3–6 h Max. excretion = 53.6 μmol	(21)
Maqui berry extract Delphinol^®^	Delphinidin-3-O-glucoside (DG) and cyanidin-3-O-sambubioside (CS) found in plasma. Gallic and protocatechuic acids were found below limit of quantification	DG plasma t_max_= 1 h, C_max_= 21.39–63.55 nmol/L. CS plasma t_max_= 2 h, C_max_= 3.46–12.09 nmol/L. GA and PCA below limit of quantification (DG: < 15.87 nmol/L; PCA: < 284.19 nmol/L).	NA	(22)
Black currant extract	Delphinidin-3-O-rutinoside, cyanidin-3-O-rutinoside and their metabolites protocatechuic acid and gallic acid were monitored	Delphinidin-3-O-rutinoside: plasma t_max_= 1.5 h, C_max_= 8.6 nM; max Cyanidin-3-O-rutinoside: plasma t_max_= 1.4 h, C_max_= 9.8 nM; Protocatechuic acid: plasma t_max_= 1 h, C_max_= 25.7 nM Gallic acid: plasma t_max_= 1 h, C_max_= 12.9 nM	Delphinidin-3-O-rutinoside urine excretion rate at 0–2 h= 20 nmol/h, Excreted % (48 h): 0.040 Cyanidin-3-O-rutinoside max. urine excretion rate at 0–2 h= 22.2 nmol/h, Excreted % (48 h): 0.048 Protocatechuic acid; max. urine excretion rate at 0–2 h= 136.4 nmol/h, Total excretion (48 h): 2,492 nmol Gallic acid; max. urine excretion rate at 0–2 h= 20.5 nmol/h, Total excretion (48 h): 235 nmol	(23)
Purple potato extract rich in methoxysubstituted monoacylated anthocyanins	ACN-associated metabolites found in plasma and urine: 4-hydroxybenzoic acid, vanillic acid, isoferulic acid-3-O-glucoronide	4-Hydroxybenzoic acid: plasma t_max_=1.4 h, C_max_=214.3 nM Vanillic acid: plasma t_max_=1.4 h, C_max_= 91.5 nM Isoferulic acid-3-O-glucoronide: plasma t_max_=2.2 h, C_max_= 161.2 nM;	4-Hydroxybenzoic acid urine t_max_=4–8 h, Max. excretion = 1,791.7 nM Vanillic acid urine t_max_=0–4 h, Max. excretion = 676.7 nM Isoferulic acid-3-O-glucoronide, urine t_max_= 4–8 h, Max. excretion = 345.6 nM	(24)
Powdered plant extract rich in cyanidin and delphinidin	Fourteen metabolites of hippuric acid, hydroxybenzoic acid, hydroxycinnamic acid, and a methylation and glucuronidation metabolite of delphinidin (malvidin-glucuronide) were detected in plasma, being 4-methoxybenzoic acid-3-glucuronide, 4-hydroxy-3-methoxybenzoic acid, and 4-hydroxyhippuric acid	4-Methoxybenzoic acid-3-glucuronide t_max_= 0.5 h C_max_= 37.8 nM; 4-hydroxy-3-methoxybenzoic acid t_max_= 1.0 h C_max_= 84.1 nM; 4-hydroxyhippuric acid acid t_max_= 1.0 h C_max_= 40.3 nM.	NA	(25)

^a^Not detected at the beginning of the study. NA, Not available.

### 2.2. Analysis of anthocyanin composition

To standardize the quantities of the familiar sources of ACNs, the Database on Polyphenol Content in Foods (26), Phenol-Explorer, and FoodData Central (27) were explored to obtain data on the concentrations of each ACN found in fruits, supplements, juices, and extracts used in CT. When concentrations found in databases did not provide sufficient information, the composition of the specific source was searched in the literature.

### 2.3. Literature review for clinical trials using anthocyanins in obesity-related inflammation

The data obtained and analyzed from each clinical study included in this report were the composition and individual doses of ACNs from various sources and the most common molecular markers for characterizing obesity and obesity-associated inflammation (lipid metabolism, glucose, cytokines, and adipokines).

#### 2.3.1. Inclusion criteria

A search restricted to years 2007–2022 of CTs that contained the words “anthocyanin” or “anthocyanins” in the title and the words “obesity” or “inflammation” in any section of the text in three databases was carried out, finding 266 entries in Scopus, 201 in Web of Science, and 250 in PubMed. Subsequently, duplicated entries were removed to refine the search, and exclusion criteria were established to obtain data with fewer sources of variability and avoid ambiguity.

#### 2.3.2. Exclusion criteria

A thorough search in titles and abstracts allowed to exclude non-blinded trials, single-dose trials, and trials that tested non-ACN mixtures or mixtures whose ACN dose could not be defined. Full-text reading allowed to exclude trials that tested ACN sources whose single ACN compositions were not reported in at least one peer-reviewed article, trials that did not measure inflammation-associated markers, and trials in which markers could not be quantified relative to placebo control. Forty-nine articles were analyzed to extract the source of ACN interventions, individual ACN composition, dose, and change of obesity-related inflammation markers.

#### 2.3.3. Dose–effect correlations analysis

Obesity-related inflammation markers were expressed as fold-change relative to placebo controls. Using the 10 most commonly measured markers and single ACN doses for each analyzed trial, correlations were calculated using the Metaboanalyst algorithm web-based platform (5.0) (28).

#### 2.3.4. Heatmap and principal components analysis

**Figure 2:**
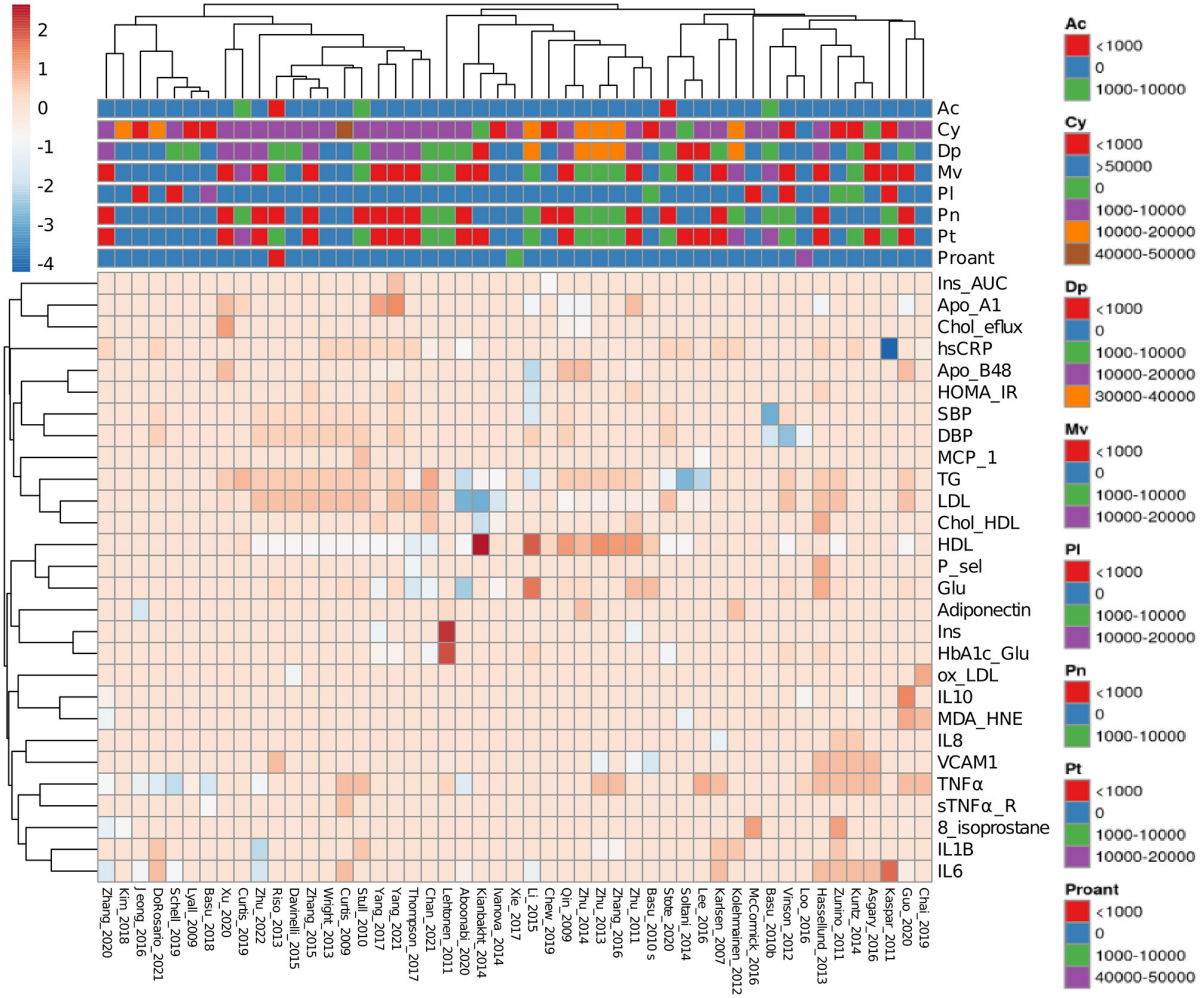
Heatmap of reviewed clinical trials measuring obesity-related inflammation parameters. Data from clinical trials were collected as a percentage of change relative to placebo controls. Anthocyanin doses are expressed as total mg consumed during the entire trial. Spaces showing absent acylated anthocyanins or proanthocyanidins mean that they were not determined. Ac, Acylated anthocyanins; Cy, cyaniding; Dp, delphinidin; Mv, malvidin; Pl, pelargonidin; Pn, peonidin; Pt, petunidin; Proant, proanthocyanidins. Complete abbreviations are shown in Supplementary Table 1.

Percentages of change relative to placebo controls and total doses were calculated as follows: (daily doses) ^*^ (study duration). The results are presented in a heatmap (Figure 2). Studies made in healthy subjects' cohorts were subtracted from data in Figure 2 to identify the main factors describing data from CTs and extract other possible correlations. Thirty-nine studies comprising the whole dataset and subgroups described in Sections 3.2.3–3.2.5 were included in performed PCAs. The online platform Clustvis was used for heatmap and PCA generation (29).

### 2.4. Literature review for pre-clinical studies using single anthocyanins in obesity-related inflammation

Studies investigating the properties of individual ACNs against obesity-related inflammation in the last 5 years (2017–2022) were reviewed. Only cell and animal models were analyzed since results from CTs using single purified ACNs were unavailable. From this search, 19 studies comprising 26 single ACN tests were analyzed.

## 3. Results and discussion

### 3.1. Absorption and pharmacokinetics of anthocyanin-rich matrices

Regarding maximum anthocyanin absorption, most studies reported that the required time to reach the maximum concentration (t_max_) for ACNs was approximately 1.5 h (23). Plasma concentrations of intact ACNs ranged from 0.2 to 63.5 nM in plasma (18, 22). Among the variability sources in metabolomics and pharmacokinetics analyses, several variables significantly influence the respective results. For instance, physicochemical properties of the delivery matrix might cause chemical interactions of anthocyanins and other compounds. Most of the studies in Table 1 used aqueous extracts or juice; however, two studies administered steamed purple sweet potato and homogenized raspberries, respectively (18, 24). In plant foods, anthocyanins and other phenolics are present in free form but also bound covalently to macromolecules such as fiber. The binding of phenolics with dietary fiber modifies the released form of bioaccessible phytochemicals from the food matrix and the actual amount of phenolics and their metabolites absorbed in the intestine and detected in plasma and urine (30). Moreover, ethanol present in anthocyanin-rich beverages exerts crucial effects on anthocyanin intestinal bioavailability, favoring their transport across intestinal epithelia (13). That contributes to the partial explanation of the variability of maximum anthocyanin concentration in plasma in studies using the same anthocyanin source; for instance, in a study by Marques et al. (31), they identified both plasma and urine samples in a study where volunteers consumed a blackberry puree with or without ethanol. In that study, the plasma concentration of anthocyanin metabolites was approximately 10 times higher than the parent anthocyanin concentration. Moreover, 12% ethanol in the matrices further increased the plasma concentration of anthocyanins from 2.0 to 2.8 ng/mL, and anthocyanin conjugates from 5.8 to 8.9 ng/mL (31). Plasma and urine preparation also accounts for part of the variability in pharmacokinetics studies of anthocyanins. While studies such as Fernandes et al. (19), Kalt et al. (20), and Castello et al. (21) enriched the anthocyanin and phenolics fraction through solid-phase extraction using C18 cartridges from plasma and urine, the study by Ludwig et al. (18) used acidified urine and plasma vacuum-dried and reconstituted in acidified methanol/water before injection in the mass spectrometry equipment. The greatest plasma concentration was achieved for delphinidin-3-O-glucoside by a single dose of 1,000 mg Delphinol^®^ at 1 h (22).

However, even greater concentrations of ACN-related metabolites were found in plasma. For instance, in a study where 250 mL of aqueous extract of red grape pomace providing 2.6 mmol ACNs were administered to healthy men, after approximately 3.5 h, a maximum concentration of 512.4 nM was found for methylpyrogallol-sulfate and 408.5 nM for protocatechuic acid-3-sulfate (21). The dosing and administration scheme plays a relevant role in the maximum concentrations detected in plasma. A study by Kalt et al. (20) shows that a single dose of ACNs promoted a higher concentration than the same dose divided into three throughout the day. Regarding ACN excretion, parent molecules and their metabolites have been detected and quantified in urine. The maximum concentration of ACNs and metabolites is usually greater in urine than in plasma, and it is important to notice that pelargonidin has been detected in urine from healthy volunteers with no consumption of ACN-rich foods (32), evidencing that ACN bioavailability depends on several metabolites that must be considered for determining their bioactivities. For the literature considered in this review, the maximum urine concentration of metabolites was found in a trial where 350 g of steam-cooked potato providing 152 mg of anthocyanins to healthy men, with a urine concentration of 1.7 nM for 4-hydroxybenzoic acid (t_max_ = 4–8 h), 676.7 nM vanillic acid (t_max_ = 0–4 h), and isoferulic acid-3-O-glucuronide (t_max_ = 4–8 h). In this regard, several ACN metabolites have been identified in plasma and urine in human studies, where the most frequently reported are gallic acid, cyanidin-O-glucuronide, protocatechuic acid, methylpyrogallol-sulfate, 4-methoxybenzoic acid-3-glucuronide, 4-hydroxy-3-methoxybenzoic acid, 4-hydroxyhippuric acid, vanillic acid, isoferulic acid-3-O-glucuronide, catechol, 4-hydroxyphenyl acetic acid, hippuric acid, phloroglucinol, and phloroglucinaldehyde (18, 19, 21–25).

In addition, to the evidence showing ACN bioavailability, the detection process of such information must be carefully noticed. There are several pitfalls and limitations when discussing pharmacokinetic parameters from polyphenols and other phytochemicals. When comparing results from different studies, it is important to consider the factors influencing the variability of reported data. For instance, sample preparation of biological fluids before analysis of polyphenolic species by mass spectrometry impacts the quantification and detection of some chemical species, as well as inner parameters from the mass spectrometry equipment such as the ionization type and source, detection method, and chromatographic column. In general, for pharmacokinetics analyses of anthocyanin and phenolics by mass spectrometry, the most common and reliable approach is a targeted mass spectrometry analysis with multiple reaction monitoring using positive ionization. Even though some studies include both positive and negative ionization in their studies to cover as many compounds as possible (33), it has been reported that even interlaboratory experimentation using the same approach, reagents, and parameters results in inevitable variability in identifying phytochemicals (34). Moreover, some physiological conditions, such as inflammations, have been demonstrated to promote variability in the pharmacokinetics of some drugs (35).

Concerning the bioavailability and pharmacokinetics of ACNs, it is relevant to highlight that the plasma concentrations of ACN metabolites are generally higher than those of the unmetabolized ACNs. As a significant number of mechanistic insights of bioactive compounds such as anthocyanins come as a result of *in vitro* and *in vivo* models, future studies focusing not only on evaluating parent molecules but also on their metabolites would help elucidate the indirect mechanisms of action by which anthocyanins promote health benefits. To investigate whether there is a relationship between individual ACNs and the reported effects, data on ACN composition and doses were collected or estimated from CTs investigating ACN interventions in obesity-related inflammation. Data were analyzed to dissect whether objective correlations could be obtained.

### 3.2. Analysis of anthocyanin doses and effects in clinical trials

#### 3.2.1. Anthocyanin compositions reported in clinical trials interventions are highly variable

Database on Polyphenol Content in Foods (26), Phenol-Explorer, and Food Data Central (27) contain the composition of multiple polyphenol sources. However, as shown in Table 2, the content of ACNs differs substantially in some studies. This could be attributed to ACN concentrations depending on factors including specific cultivar, year, and zone of ripping or even to the used extraction and detection method as discussed in the previous section. For example, to report the red raspberry composition, Ponder et al. (47) extracted ACNs using acidified methanol and identified/quantified them by LC-DAD (high-performance liquid chromatography combined with diode array detection). On the other hand, some extractions for Phenol-Explorer data included other phenolics thus using several consecutive ethyl acetate and methanol extractions, and identifications/quantifications were made using LC-DAD and LC-MS (high-performance liquid chromatography combined with electrospray ionization mass spectrometric detection). Thus, ACN determination based on literature reports is challenging; however, standardized sample preparation and detection methods in future studies should provide more homogeneous and comparable results easier to interpret. For example, Phenol-Explorer data provide valuable information about the phenolic composition and total ACN content of food sources that can be complemented as new single ACN determinations are reported. Therefore, identifying individual ACNs and their concentration in CTs allows one to analyze and compare their effects to get more information about pure compounds' effects and synergistic or antagonistic behaviors. In addition, purified and fully characterized mixtures of anthocyanins could contribute to understanding their efficacy and mechanisms of action. Medox^®^ dietary supplement is the most common ACN extract that contains mostly delphinidins and cyanidins with minor concentrations of petunidins, malvidins, and peonidins and does not contain pelargonidins. Medox^®^ is among the best characterized and standardized ACN-rich products (see Table 2).

**Table 2:** Summary of the anthocyanins (ACN) content reported in sources cited in this review.

**Source**	**ACN**	**Min**	**Max**	**Average**	**Dry average**	**%**	**Phenol-Explorer average**	**References**
Caucasian Whortleberry tablets,	DpGlu			3.25				(36)
*Vaccinium arctostaphylos*,	PtGlu			2.50				
mg/100 g FW	Mvglu			4.00				
Dried strawberries,	PgGlu					84.00	62.36	(37)
*Fragaria x ananassa*	PgRut					9.00	1.32	
% in ACN extract from five cultivars	CyGlu					7.00	4.10	
Dried Acai,	CyGlu				4.94			(38)
*Euterpe oleraceae Mart*., mg/100 g DW	CyRut				17.90			
Blueberries, Vaccinium repository	MvGal/PtGlu	5.50	84.10	39.10			21.43/11.2	(39)
	MvGlu/PtAra	0.10	61.70	28.10			26.06/3.54	
Data from 80 genotypes mg/100 g FW	DpGlu/CyGal	0.10	118.00	27.50			15.17/7.99	
	DpGal	0.90	62.80	25.60			16.14	
	PtGal/CyAra	7.70	59.30	21.90			2.76/4.03	
	MvAra	6.40	52.00	19.80			7.89	
	PtGlu	1.40	38.20	16.60			11.20	
	CyGlu/DpAra	5.20	39.10	15.80			7.50/7.32	
	PtAra/PnGal	4.80	30.10	14.80			3.54/2.76	
	Ac	0.10	34.00	11.60				
Purple carrot, *Daucus carota*, mg/100 g FW	CyXylGluGal			1.30				(40)
	CyXylGal			12.50				
	SinCyXylGluGal			1.10				
	FerCyXylGluGal			18.20				
	CouCyXylGluGal			6.40				
Red grape/Bilberries juice, *Vitis vinifera* 80%, *Vaccinium myrtillus* 20% in 330 ml	Pn			189.21				(41)
	Cy			66.50				
	Dp			92.77				
	Pt			96.00				
	Mv			395.54				
Purple potato, *Solanum tuberosum*, % in DW	CyCouRutGlu	4.6	7.6			6.27		(42)
	PtCouRutGlu	57	68.4			63.13		
	PgCouRutGlu	ND	10.1			2.18		
	PnCouRutGlu	11	15.9			13.22		
	PnFerRutGlu	1	2.2			1.58		
	PtCafRutGlu	3.5	6.1			4.67		
	PtFerRutGlu	2.8	4.5			3.38		
	MvCouRutGlu	1.3	4.8			2.77		
Queen Garnet Plum, *Prunus salicina Lindl*. in 100 ml of juice	CyGlu			200.00				(43)
	CyRut			30.00				
Black raspberry capsules, *Rubus occidentalis*, mg/100 g DW	Cy			100.00				(44)
	Pl			4.00				
	Proant			19.50				
Black soybean, *Glicine max*, % in ACN extract	CyGlu					68.30		(45)
	DpGlu					25.50		
	PtGlu					6.50		
Chokeberries, *Aronia melanocarpa*, Content in 300 ml of juice + 3 g of powder	CyGal			647.00				(46)
	CyGlu			30.00				
	CyAra			310.00				
	CyXyl			26.00				
	Proant			745.00				
Red raspberries, *Rubus ideus L*., mg/100 g FW	CyGlu			45.42			14.89	(47)
	PlGlu			9.69			1.65	
	DpGlu			34.43			0.21	
Black currant, *Ribes nigrum*, data from 11 cultivars mg/100 g FW	DpRut	40.17	137.66	92.03			304.91	(48)
	DpGlu	7.80	42.22	23.93			86.68	
	CyRut	25.77	129.09	70.48			160.78	
	CyGlu	0.58	20.82	7.72			25.07	
Bilberries, *Vaccinium myrtillus*, % in Myrtocyan^®^ extract	DpAra					4.32		(49)
	DpGlu					5.81		
	DpGal					5.04		
	CyAra					2.19		
	CyGlu					3.42		
	CyGal					2.75		
	PnAra					0.22		
	PnGlu					1.31	37.47	
	PnGal					0.34		
	PtAra					1.08		
	PtGlu					3.67		
	PtGal					1.89		
	MvAra					0.81		
	MvGlu					3.35		
	MvGal					1.27		
Delphinol^®^ from maqui, *Aristotelia chilensis*, mg/100 g FW	DpSamGlu			46.40				(50)
	DpDiGlu			23.70				
	CySamGlu/CyDiGlu			18.70				
	DpSam			14.20				
	DpGlu			17.10				
	CySam			8.90				
	CyGlu			8.60				
Medox^®^ from bilberries/blackcurrant, *Vaccinium myrtillus* and *Ribes nigrum*, % per capsule	CyGlu					11.00		(51)
	CyGal					11.00		
	CyAra					11.00		
	DpGlu					19.33		
	DpGal					19.33		
	DpAra					19.33		
	PtGlu					0.83		
	PtGal					0.83		
	PtAra					0.83		
	PnGlu					0.83		
	PnGal					0.83		
	PnAra					0.83		
	MvGlu					1.00		
	MvGal					1.00		
	MvAra					1.00		
	CyRut					0.50		
	DpRut					0.50		
Artemis int B0120034 from Elderberry, *Sambucus nigra*, Content per capsule	Cy			125.00				(52)
CherryActive^®^ concentrate from Tart montmorency, *Prunus cerasus*, content in 30 ml of concentrate	CySop			2.20				(53)
	CyGluRut			105.70				
	CyGlu			1.70				
	CyRut			46.60				
	PnGlu			9.10				
	Cy			0.50				
Artemis Int 24198000 from chokeberry, *Aronia melanocarpa*, in 500 mg of extract	CyGal			6.27				(54)
	CyAra			2.31				
	CyGlu			1.12				
	Proant			23.40				
Ocean Spray cranberries, *Vaccinium oxycoccus*, in 450 ml of juice	CyAra			1.52			4.47	(55)
	CyGal			1.78			8.89	
	CyGlu			0.07			0.74	
	PnAra			0.80			9.61	
	PnGal			1.77			22.02	
	PnGlu			0.22			4.16	

The “/” symbol in cited references means the chromatographic separation was not able to distinguish between both compounds; for Phenol-Explorer, reported values correspond to individual compounds. The first column indicates common and scientific names of the sources and units in which minimum (Min, 3rd column), maximum (Max, 4th column), and average (5th column) contents of single ACNs (2nd column) are expressed. The majority of contents are reported as mg/100 g of fresh weight; when contents were expressed as mg/100 g of dry weight or percentages of total ACNs, they were listed in 6th or 7th column, respectively. When available, contents reported in Phenol-Explorer were listed in the 8th column in the same units as the corresponding 3rd−8th column. The name of individual anthocyanins is formed by two letters for the aglycone part and three for the substituents in 3-O or 5-O position; further substituents to carbohydrate structure are also shown by three letters. Ac, Acylated anthocyanins; Cy, cyanidin; Dp, delphinidin; Mv, malvidin; Pg, pelargonidin; Pn, peonidin; Pt, petunidin; Ara, arabinoside; Gal, galactoside; Glu, glucoside; Rut, rutenoside; Sam, sambubioside; Sop, soporoside; Xyl, xyloside; Caf, caffeic acid; Cou, coumarinic acid; Fer, ferulic acid; Sin, sinapic acid; Proant, proanthocyanidins.

Regardless, there are many other ACN-rich nutraceuticals, e.g., the Aronia berry (Chokeberry) powder (Product code 24198000 from Artemis International), whose composition was considered as that reported by Cujić et al. (54) and is shown in Table 2. Since data obtained from trials using purified ACNs cannot be directly compared with those using uncharacterized sources, the reported ACN compositions in Table 2 were used to estimate individual ACN quantities in CTs where quantifications of ACNs were unavailable.

#### 3.2.2. Lipid metabolism-related markers are the most represented in the reviewed literature

To collect recent data regarding the effect of ACNs on obesity-related inflammation, the literature was reviewed. ACN effect has been evaluated on healthy men and women, older adults, and volunteers with obesity, type II diabetes, hypertension, hyperlipidemia, non-alcoholic fatty liver disease, and metabolic syndrome. This review included CTs that evaluated inflammation markers altered by obesity conditions in volunteers receiving fruits, extracts, juices, or tablets rich in ACNs. The CTs were conducted for up to 24 weeks with a maximum daily ACN dose of 1,380 mg. The main objective of these interventions focused on evaluating the beneficial effects of ACNs on anthropometric parameters (e.g., weight or BMI) and plasmatic markers (e.g., glucose, cholesterol, or C-reactive protein). However, the specific anthocyanin effect remains unclear.

To graphically depict the most represented and modified markers studied in the reviewed literature, data from CTs can be observed in Figure 2. This heatmap shows how the distinct ACN sources changed the measured markers in the selected studies. Among them, five reported the quantity of the ACN source (fruit or extract) without specifying ACN determination (56–60), 19 reported the total ACN content of applied doses (61–79), eight reported total and individual ACN concentrations (40, 41, 44–46, 52, 53, 55), and 17 used the Medox^®^ dietary supplement (51, 80–95).

Measurements of markers directly related to lipid metabolism are the most represented data, followed by inflammation markers such as interleukins 6 and 10 (IL-6 and IL-10) and tumor necrosis factor α (TNFα). However, in some studies, some oxidative stress markers such as oxidized low-density lipoproteins (oxLDL), malondialdehyde/4-hydroxynonenal (MDA/HNE), and 8-isoprostane were also reported. Regarding administered ACNs, it can be observed that there is a great variation in part due to acylated ACN and proanthocyanidin content being heterogeneous. As the color code indicates, determinations for C-reactive protein (CRP) (67), high-density lipoprotein (HDL) (79), and insulin (59) showed the most significant changes.

As basal concentrations of analyzed markers varied among cohorts, data from healthy subject cohorts were not included in Figure 2 but were analyzed separately.

#### 3.2.3. Clinical trials on healthy subjects reported petunidin may increase IL-6 levels

The significant changes found in healthy subject cohorts were interleukin 8 (IL-8, −20.42%), IL-6 (-18 to +18%), CRP (-175%), IL-10 (+173%), and LDL (−24.8%) (41, 52, 53, 66, 67, 70, 81, 83, 85). Kaspar et al. (67), Guo et al. (81), and Ivanova et al. (66) reported the most relevant effects. Kaspar et al. showed that ACNs from cooked purple potatoes (297 mg daily for 6 weeks) produced a 175% reduction in CRP levels and an 18% increase in IL-6 levels in young men (18–40 years old). As this trial was the only one that reported an increase in IL-6 levels, it is possible that the high petunidin content (42) in purple potatoes may be connected to that change. Guo et al. showed that Medox^®^ capsules (320 mg of ACNs daily for 2 weeks) produced a 173% increase in IL-10 levels and an 18% reduction in IL-6 levels in a cohort including predominantly young women (18–35 years old) (81). Ivanova et al. (66) showed that dwarf elderberry (*Sambucus ebulus* L.) infusion (4 mg cyanidin daily for 4 weeks) produced a decrease in LDL and TG levels (24.8 and 14.15%, respectively) in a cohort including mainly women. The reported ACN dose by Ivanova et al. (66) was lower than in other trials, yet it produced beneficial effects, indicating that infusions may be an effective way to enhance the bioavailability of ACNs.

To sum up, data from the subgroup with healthy cohorts reporting decreases for TG were < 15%, possibly due to basal levels (between 64 and 96 mg/dL) not being as high as those found in subjects with obesity, hyperlipidemia, or other pathological conditions (between 130 and 304 mg/dL). In the same way, baseline HDL levels varied between 42 and 76 mg/dL in healthy subjects but between 44 and 48 mg/dL in subjects with obesity, hyperlipidemia, or other pathological conditions.

#### 3.2.4. Delphinidin was more consistently associated with improved high-density lipoprotein levels

**Figure 3:**
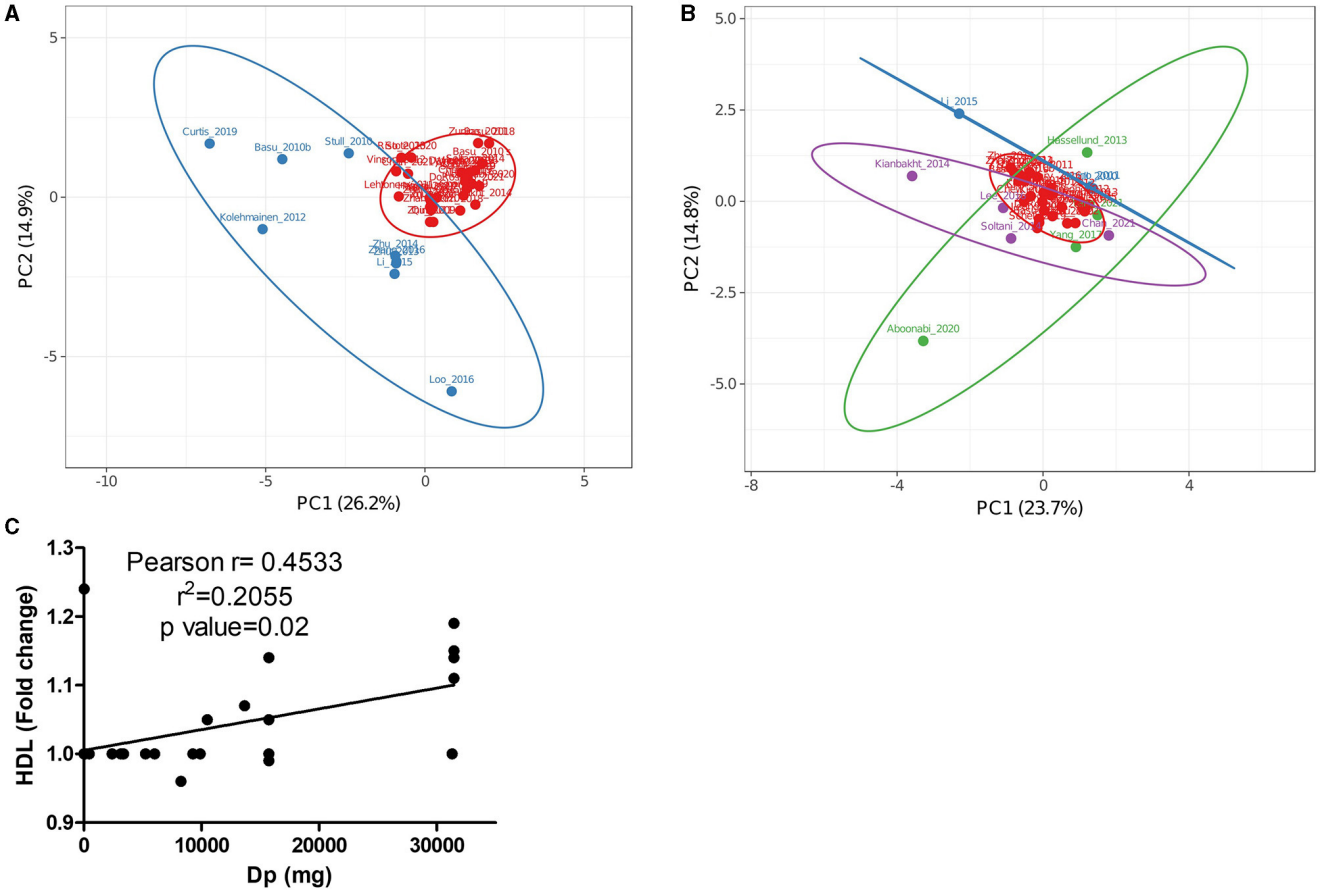
Principal component analysis (PCA) of data from Figure 2 studying cohorts with pathologies including **(A)** or not **(B)** single anthocyanin doses as factors. **(C)** Correlation of HDL with delphinidin doses in trials included in **(A, B)**. **(A)** The Red group represents trials with low variance and the blue group represents trials with high variance. **(B)** The red group represents trials with low variance, the blue group represents trials with high variance in **(A, B)**, the green group represents trials with high variance that used Medox^®^, and the purple group represents trials with high variance that used uncharacterized or partially characterized anthocyanin sources.

Principal components analysis (PCA) is a technique that allows the reduction of dimensions of datasets containing multiple features per observation. Thus, the PCA technique was used because the obtained dataset contained many variables related to each study. Figure 3A shows a PCA from 39 of the 49 included studies in Figure 2, where data from calculated ACN doses and shifts produced in measured markers were studied. Two principal components explained 41.2% of the variance in this analysis. From the PCA, it was observed that most of the studies with high variances (blue group in Figure 3A) were those in which the highest ACN doses among included studies were used (46, 56, 64, 69, 84, 91, 93, 95). To avoid the doses being the main variable describing variance, PCA was performed excluding ACN doses. Figure 3B, describing 38.5% of the variance, showed that only three of the studies with high variance in Figure 3A had high variance (blue group in Figure 3B) when ACN doses were excluded from the analysis (60, 74, 84). Hypothesizing that high variance in both PCAs should reflect the accurate determination of concentrations and their effects, details of CTs in such cases are mentioned. Trials by Zunino et al. (60) and Stull et al. (74) reported no changes for most of the markers collected in this review in response to ACN interventions [only Stull et al. (74) found insulin sensitivity increased by 17.3%]. On the other hand, Li et al. (84) found important changes for several markers [−7.9% low-density lipoprotein (LDL), +19.4 HDL, −23% triacyl glycerides (TG), −8.64% TNFα, −31.58% IL-6, −16.5% apolipoprotein B-48 (ApoB-48), −11% apolipoprotein C-III (ApoC-III), −23.62% adiponectin)]. Daily ACN dose and time of interventions were highly different (Li: 320 mg/24 weeks, Stull: 668 mg/6 weeks, and Zunino: 422.4 mg/3 weeks) and evidenced in the PCA through the modified markers.

Besides the studies with high variances in Figures 3A, B, the highest variances in Figure 3B were found in trials using Medox^®^ (green group) (80, 82, 87, 88) and interventions with cyanidin, malvidin, and petunidin, as main components (purple group) (45, 63, 73, 79). Trials by Hassellund et al. (82), Yang et al. (87, 88), and Chan et al. (63) had almost the same load for the PC1 comprising primarily LDL, HDL, and TG levels corresponding also with similar total daily ACN doses (640 mg/4 weeks for Hassellund trial, 320 mg/4 weeks for Yang trials and 400 mg/4 weeks for Chan trial). The PC2 in Figure 3B comprised mainly glucose, HDL, and TNFα levels; however, interventions produced similar changes (< 10%) for measured markers in studied cohorts [prehypertensive by Hassellund et al. (82), prediabetic by Yang et al. (87) and type-2 diabetic by Chan et al. (63) found]. Although Chan et al. (63) found a significant reduction in CRP levels (31.81%), this effect was not greatly reflected in PCA analysis. Interestingly, the four studies (63, 82, 87, 88) shared interventions using predominantly delphinidin but only the trial using the cohort with diabetes found a significant CRP drop. This fact may point toward an important function for CRP as a sensitive marker for future research addressing the ACN effect in cohorts with diabetes.

Intervention times among the trials using uncharacterized ACN sources with positive values for PC1 ranged from 4 to 8.6 weeks; however, total ACNs administered through the whole interventions varied from 63 to 1,766 mg. Doses did not correlate with the found variance since the study by Kianbakht et al. (79) had the lowest dose but showed the greatest variance. While Soltani et al. (73) and Kianbakht et al. (79) tried *Vaccinium arctostaphylos* extract tablets (delphinidin and malvidin glucosides as main components) on cohorts with hyperlipidemia, Lee et al. (45) tried black soybean extracts (cyanidin glucoside as main component) on subjects with obesity and overweight. The most crucial difference between the trials may be the cohorts since subjects in Kianbakht et al. (79) study had a higher baseline for LDL and TG levels (≈165 and 300 mg/dL, respectively) than subjects in Lee et al. (45) and Soltani et al. (73) studies (≈118 and 195; and 126 and 209 mg/dL, respectively). This difference may explain why Kianbakht et al. using a low dose of ACN tablets (2.45 mg daily by 8.6 weeks) found a bigger impact on lipid markers.

The results among the studies with higher variances in PCAs reflected that concentrations of single ACNs in the database could not be completely correlated with observed effects. Moreover, certain differences suggested that greater changes in markers were associated with their higher initial baseline and severity of the diseases in the studied cohorts.

To evaluate the effect of increased baseline levels, the correlation of the most common obesity-related inflammation markers with single ACN supplementation doses was assessed (Supplementary Figure S1). Although the performed analysis showed that HDL, LDL, and TG levels were the most modified values by all ACNs, HDL level was the only parameter significantly correlated with delphinidin doses (Figure 3C). Although the correlation coefficient was low (R^2^ = 0.2055, *p* = 0.02), the tendency found in our dataset suggests that further studies using delphinidin-enriched extracts or purified delphinidins may clarify their role as anti-obesogenic compounds. Specifically, observations made from trials with diabetic subjects encourage future research to dissect the effects of delphinidin and the mechanisms of action in these cohorts. The poor correlation found for most ACNs may be due to several factors, namely, the influence of the ACN-free fraction, synergic or additive effects from other ACNs, and modified bioavailability among different matrices.

#### 3.2.5. Observations about doses and effects from subgroup analyses

##### 3.2.5.1. Increased medox^®^ efficacy was found in advanced inflammatory conditions

**Figure 4:**
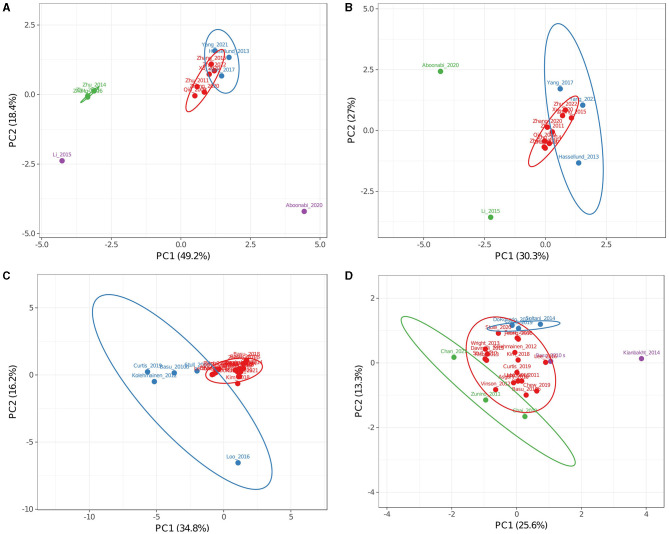
Principal component analysis of clinical trials addressing obesity-related inflammation in Medox^®^ **(A, B)** or uncharacterized **(C, D)** subsets. Analyses **(A, C)** included single anthocyanin doses as factors while **(B, D)** analyses did not. Colors are used to separate groups of trials discussed in the text. **(A)** The red group represents trials with low variance, the blue group represents trials with moderate variance, and the green and purple groups represent trials with high variance. **(B)** The red group represents trials with low variance, the blue group represents trials with moderate variance in **(A, B)**, and the green group represents trials with high variance in **(A, B)**. **(C)** The red group represents trials with low variance, and the blue group represents trials with high variance. **(D)** The red group represents trials with low variance, the blue group represents trials with positive values for PC2, the green group represents trials with negative values for PC1 or PC2, and the purple group represents trials with positive values for PC1.

As comparisons made among the complete dataset did not produce conclusive data and doses of single ACNs should be homogenous in CTs in which only Medox^®^ was used, a PCA was performed on a subset of these CTs. Figures 4A, B shows PCAs for complete (including dosing, Figure 4A) and only markers (ACN doses not included, Figure 4B) data. When dosing was incorporated into the analysis (Figure 4A), PC1 comprised predominantly ACN doses and described 49.2% of the variance, while PC2 comprising TG, LDL, and CRP levels explained 18.4%. As observed in the global analysis of CT, including dosing, studies from the research group of Li, Zhang, Zhu, and co-workers (91, 93, 95) showed high variance (Figure 4A, green group) in the opposite way to the trial in which the lowest ACN quantity was tested (purple dot) (80). CT with moderate variance (82, 87, 88) also appeared separated from the central cluster in both PCAs (blue group in Figures 4A, B). When ACN dosing data were excluded from this analysis (Figure 4B), only five trials conserved high variance (blue and green groups) (80, 82, 84, 87, 88). When dosing was not included in PCA, two main factors explained 54.6% of the variance; PC1 was mainly affected by TG, LDL, and CRP levels, while PC2 was by HDL, glucose, ApoA1, and TNFα levels. A CT conducted by Hassellund et al. (82) reported that 640 mg of Medox^®^ administered daily for 4 weeks to prehypertensive non-dyslipidemic subjects modified HDL (+5.08%) and glucose (+2.76%). As other markers were unaffected in the Hasselllund study, increased glucose level determined its main variance. On the other hand, Yang et al. (87, 88) explored the effect of Medox^®^ supplementation in prediabetic patients and found moderate changes in several markers (−5.52 and 0% LDL; −2.86 and 0% TG; −2.56 and 0% CRP; −5.66 and 0% glucose). Similarly, Yang et al. (88) also analyzed ApoA and ApoB and found moderate changes (+6.35 and −6% average, respectively) compared with the modulation reported by Li et al. (84). Li et al. (84) found that ApoB levels decreased by 16.5% in diabetic subjects treated during 24 weeks with Medox^®^ supplementation. Li et al. (84) also found greater changes in other markers than in prediabetic cohorts by Yang et al. (87, 88) (+19.4 vs. −1.3% HDL and −23.0% vs. −2.9% TG). Differences between the results of the mentioned trials may be attributed to the status of diabetes progression or the treatment duration. Future CTs including stratification of the diabetes stages and Medox^®^ supplementation for 12, 18, and 24 weeks would clarify the impact of each factor.

Interestingly, results reported by Aboonabi et al. (80) from subjects with metabolic syndrome for 4 weeks were found separated from the rest of the CTs when dosing was included in the analysis or when it did not. In contrast to all other cohorts, including mainly hypercholesterolemic, prediabetic, or dyslipidemic individuals in the subset of Medox^®^ trials, high changes found after the supplementation in this cohort (−36.4% low LDL, −27.0% TG, −31.0% TNFα, −42.5% CRP, and −12.5% glucose) may be due to a more advanced inflammatory state. From this observation and the differences between prediabetic and diabetic cohorts, it can be inferred that ACN supplementation may be more useful in critical health conditions where the inflammatory response is exacerbated.

##### 3.2.5.2. Clinical trials using uncharacterized anthocyanin sources suggested cyanidin-derivatives reduce CRP levels

The same PCA analyses for the whole group and the Medox^®^ group were done for the subset of CTs in which sources of uncharacterized or only partially characterized ACNs were used. Two main factors described 50.9% of the variance when the analysis included dosing (Figure 4C) and 39.3% when it did not (Figure 4D). PCA in Figure 4C (blue group) shows that results from five CTs were apart from the most prominent cluster around the plot origin (46, 56, 64, 69, 74). When dosing data were not included in the analysis (Figure 4D), none of the non-clustered trials was apart from the main cluster; instead, trials by Chan et al. (63), Chai et al. (58), Basu et al. (56), Stote et al. (78), Soltani et al. (73) and Kianbakht et al. (79) appeared apart (green, blue, and purple dots in Figure 4D). This result, in contrast with that obtained from the Medox^®^ subset, suggests that no significant correlations exist between concentrations used for uncharacterized sources and their effects on measured metabolic markers.

Several observations can be made by analyzing the outliers in Figure 4D. A CT conducted by Basu et al. (56) reported that the consumption of freeze-dried blueberries during 8 weeks reduced levels of oxidized LDL (oxLDL) by 19% and malondialdehyde (MDA) by 8% in subjects with obesity and metabolic syndrome. Similarly, Chai et al. (58) found that consuming Montmorency tart cherries juice for 12 weeks reduced levels of oxLDL by 11% and MDA by 3% in older adults. Although data regarding oxLDL and MDA levels grouped both trials in PCA, no similarities were found in ACN composition [primarily cyanidin for Chai et al. (58) and petunidin for Basu et al. (56)] or dose [≈7-fold higher for Basu et al. (56)]. Besides, Chai et al. (58) found a 25% decline in CRP levels, very similar to findings by Zhang et al. (91) and Chew et al. (55) (21.6 and 22%, respectively). From studies that found similarly modified CRP levels to the trial by Chai et al. (58), the result by Chew et al. (55) stands out since they tried cranberry juice for 8 weeks in subjects with obesity and metabolic syndrome. Although Chew et al. reported a lower total ACN dose (≈20-fold), both trials tested juices with cyanidin glycosides as main ACNs suggesting that CRP levels can be decreased even with low cyanidin doses.

CT conducted by Do Rosario et al. (77), Schell et al. (72), and Soltani et al. (73) presented similar positive values for PC2 (blue group, Figure 4D). Daily ACN doses and duration of intervention for each study were 201 mg/8 weeks, 224 mg/4 weeks, and 90 mg/4 weeks, respectively. Regarding selected cohorts, Do Rosario et al. (77) intervened older adults with queen garnet plum juice, Schell et al. (72) intervened adults with type-2 diabetes and obesity with red raspberries, and Soltani et al. (73) intervened subjects with hyperlipidemia with Caucasian whortleberry tablets. Although Do Rosario et al. (77) reported that only TNFα levels were reduced by ACN intervention (−30.91%), IL-6 and IL-1β also tended to be decreased similarly to levels reported by Schell et al. (72) (−38.2 for TNFα and −26.4% for IL-6). Both studies used similar daily doses with slightly different compositions (Table 2). Queen garnet plum contains mainly cyanidin glucoside and ruthenoside, while red raspberries have predominantly cyanidin and delphinidin glucosides. As the main component in both studies that reported decreased TNFα levels, cyanidin could reflect a similar mechanism in older adults and subjects with obesity. Do Rosario et al. (77) and Schell et al. (72) did not evaluate lipid profiles, and Soltani et al. (73) did not evaluate TNFα or IL-6 levels; nonetheless, neither found any change for CRP levels. This fact suggests that the correlation between cyanidin content and CRP levels in Chai et al. and Chew et al. studies is complex (55, 58). Recently, Do Rosario et al. (77) and Chai et al. (58) intervened older adults with cyanidin-rich juices; while Do Rosario et al. (20) found decreased TNFα but unchanged CRP levels, Chai et al. (58) found decreased CRP but unchanged TNFα levels. These contrasting results may reflect the issue discussed in a meta-analysis by Sangsefidi et al. (96), who reported that ACNs had no significant impact on CRP levels. Our analysis suggests that CRP levels should be determined alongside other cytokines to effectively describe inflammation in future research addressing the ACN effect.

Triacyl glyceride levels after ACN interventions for studies by Soltani et al. (73) and Stote et al. (78) decreased (16.64 and 34.55%, respectively), reaching similar levels (≈158 mg/dL). Interestingly, even when the ACN dose was ≈40-fold lower in the trial by Soltani et al. (73), they also reported decreased LDL and MDA levels (11.32 and 20.68%, respectively). Soltani et al. intervened in hyperlipidemic subjects; therefore, bigger changes in lipid markers reinforce the observation that ACN interventions substantially modulate several markers in subjects with increased baseline levels.

The CT conducted by Kianbakht et al. (79) (purple, Figure 4D) used 7.35 mg of ACNs from Caucasian whortleberry tablets daily through 60 days in hyperlipidemic subjects and substantially improved lipid markers (−37.1% LDL, +24.4% HDL, and −12.2% TG). Conversely, Chan et al. (63) (green, Figure 4D) used a significantly greater daily ACN dose (54-fold higher) from bilberry through 28 days in subjects with type-2 diabetes. They produced a shallow effect on lipid markers (+1.1% LDL, −4.4% HDL, and +5% TG). Both studies reported the total anthocyanins used, but the composition was investigated in the literature and databases (Table 2). From this comparison, it may be implied that ACNs with low dosing through long periods result in better outcomes than shorter periods with high doses. In addition, ACN bioavailability obtained with a unique high dose was found higher than the same dose divided throughout the day (20). Such difference may be explained if metabolites derived from intact ACNs show effects and health benefits, thus justifying future detailed studies addressing the effects of single ACNs and their metabolites.

A comparison between studies testing raspberries revealed that even though the ACN dose was almost 85-fold higher in the study by Schell et al. (72) using black raspberry, the results were similar to those reported by Jeong et al. (44) who used red raspberry extract as intervention. Jeong et al. (44) reported their extract was composed of cyanidin, pelargonidin, and proanthocyanidins (2.64 mg daily for 12 weeks), while Schell et al. reported quantity of the used fruits, but ACN doses (223.85 mg daily of cyanidin, pelargonidin, and delphinidin glucosides during 4 weeks) were calculated. Discrepancies may be due to variability in parameters such as specific cultivar, zone, and ripening year, which are not considered in Table 2. Differences could also be due to an increased bioavailability of the ACNs present in the capsules used by Jeong et al. (44).

Specifically, the black raspberry extract used by Jeong et al. (44) comprised a cyanidin daily dose of 2.14 mg, which produced results very similar to those reported by Li et al. and Schell et al. regarding IL-6 and TNFα serum levels (72, 84). In those studies, daily cyanidin doses were similar (105.6 and 113.8 mg, respectively), and delphinidin was predominant in the Li et al. study compared to Schell et al. (72) (185.6 mg and 86.1 mg, respectively). Since the rest of the compounds were scarce in the trial by Li et al. (84), this comparison may suggest that pelargonidin, either additively or synergistically, acts with cyanidin and delphinidin to reduce TNFα serum levels (−8 vs.−38%). Furthermore, it is important to note the treatment time because the lowest TNFα level was achieved in only 4 weeks (72) with a non-purified source of ACNs compared with 24 weeks of Medox^®^ treatment (84). In both cases, the patients presented diabetes, but Schell et al. subjects also presented obesity, possibly indicating ACNs are more relevant for TNFα levels in subjects with obesity. Results from further studies should be used to contrast these data and improve our understanding of specific markers that better display the anti-inflammatory properties of ACNs.

Specific inflammation markers (TNFα, CRP, and IL-6) were not enough represented to significantly modify distribution in PCAs; however, some of the trials that found higher changes (>20%) for TNFα (44, 62, 72, 77, 80, 89, 92), CRP (55, 58, 63, 80, 91) and IL-6 (44, 72, 84, 89, 92) also revealed significant changes for other markers such as TG, HDL, and LDL levels. However, more studies should be conducted to better examine the anti-inflammatory effect and other mechanisms of action of ACNs.

From the observed studies with more increased markers (Figure 2), only the CT reported by Lehtonen et al. (59) did not show a high variance in PCA (Figure 3B). Three of all the studies found that ACN intervention changed serum insulin concentration, and Lehtonen et al. was the only study that found an increased level (+7.4%). Therefore, the augmented marker only had a small influence on the overall data. Nevertheless, it is important to notice that only six studies among the 49 included reported insulin levels. The small representation of insulin levels supports the need for this determination in future studies investigating obesity-related inflammation to clarify involved mechanisms in the ACN effect.

## 4. Single-dose and short-term interventions in postprandial studies

Postprandial studies in which ACN ability to modify changes produced by ingestion of meals were not included in PCAs as they evaluate short-term ACN effect (maximum 1 week). However, the information provided by these CTs in which inflammatory markers were evaluated resembles, to some extent, the results obtained with more prolonged treatments. The CTs have evaluated the ACN effect after consumption of meals high in fat (25, 77, 97–99) or high in fat and carbohydrates (100–102), and also have evaluated postprandial effects after consuming only ACN-rich meals (103, 104). Particularly, the high-fat meal challenge studies have been recently reviewed (105), and protective effects on oxidative stress and antioxidant status, triacylglycerol and total cholesterol concentrations, vascular endothelial function, and inflammatory biomarkers have been identified after ACN consumption. Despite reviewed CT present heterogenous results derived from different cohorts and measured markers, the modified markers were TG with 20–35% of reduction (25, 97), glucose with 8–42% of reduction (25, 106), insulin with 11–34% of reduction (100, 104), CRP with 13–22% of reduction (102, 107), and IL-6 with 7–37% of reduction (99, 106). Furthermore, positive changes regarding vascular stiffness (98), insulin sensitivity (101), oxLDL (100), malondialdehyde (99), and expression of pro-inflammatory and antioxidant genes in peripheral blood mononuclear cells (25) among others have been found.

A deep analysis of metabolites reported in the postprandial studies and the interaction of ACNs and such metabolites with gut microbiota is out of the scope of this review. However, a detailed discussion about gut microbiota interactions with ACNs and their metabolites from animal studies has already been published (108, 109). On the other hand, studies discussing bioavailability, as those mentioned in Section 3, and analysis of reported metabolites (25, 100, 102, 107) should be addressed in future works.

## 5. Cyanidin, delphinidin, and pelargonidin consistently reduced obesity-related inflammation in pre-clinical and *in vitro* cell-based studies

To get a better understanding of individual ACN impact, studies that evaluated the effect of single ACNs were reviewed (110–127).

Among the analyzed studies, pelargonidin, malvidin, delphinidin, and cyanidin produced the most remarkable changes for inflammation- and obesity-related markers (110, 113, 118, 119, 126–128). Those works with the highest variance used aglycones (anthocyanidins) even though 9 of the 26 entries tested anthocyanins. Concentrations used in these studies were 30 μM for pelargonidin in LPS-treated HUVEC cells (118, 127); 10, 50, and 120 μM for delphinidin in SKOV3 cells, neonatal rat cardiomyocytes or HCT116 cells, respectively (119, 126, 128); 100 μM for malvidin in LPS-treated human peripheral mononuclear cells (110); and 100 μM for cyanidin in LPS-treated Caco-2 cells (113). Gan et al. also determined the efficacy of cyanidin and cyanidin glucoside on 2,4,6-trinitrobenzenesulfonic acid-induced colitis in mice. By using 200 μmol/kg, they obtained reductions very similar to those obtained in Caco-2 cells regarding TNFα, IL-1β, IL-6, and IFNγ levels. Notably, in both models, they found that the effect of cyanidin and cyanidin glucoside was not statistically different. This last observation may confirm the hypothesis that anthocyanidins and anthocyanin glucosides produce similar results. Since we did not find information to make other comparisons between anthocyanidins and their corresponding glucosides, future experimentation would shed some light on this matter.

Findings from the analyzed studies suggest that pelargonidin and delphinidin are effective disruptors of the ERK 1/2 MAP kinase signaling (118, 119, 126–128) and can exert their effects at concentrations ranging from 10 to 120 μM. Moreover, VEGF can be diminished by delphinidin or delphinidin glucoside, and the concentrations reported for this effect are quite dissimilar (from 40 to 790 μM) (117, 120). Of particular interest are the findings by Jia et al. (115) and Molonia et al. (121) since both reported that cyanidin glucoside increases adiponectin levels in mouse and SGBS human adipocytes at 103 and 10 μM, respectively. Finally, classical inflammation markers such as TNFα, IL-1β, IL-6, and NFκB were mainly measured in cyanidin-treated models, proving to be an excellent candidate for preventing inflammation (113, 116, 121–123). Although pelargonidin has also been shown to prevent LPS-induced inflammation markers in HUVEC cells, further studies are required to fully understand how this prevention occurs (127).

Although comparisons made in this review are subject to many sources of variation, our data showed that pelargonidin might have promising characteristics individually or synergistically with cyanidin. Furthermore, the comparison made in Section 3.2.5.2 between CTs (72, 84) suggested that pelargonidin could decrease TNFα serum levels synergistically with cyanidin. Pelargonidin-3-O-glucoside alone was administered to diluted human whole blood stimulated with LPS, and no effects were observed on cytokine levels except for IL-10, which increased by 18% (129), reinforcing the idea that it synergistically exerts an effect with cyanidin or other ACNs. However, the low potency of ACNs in general and specifically pelargonidin has been associated with instability in the human physiological environment, and some studies have attempted to develop delivery strategies to improve pelargonidin bioavailability (130). Therefore, encapsulation might be an alternative to deliver ACNs to exert their beneficial effects effectively.

On the other hand, cyanidin is the most abundant ACN in several fruit and vegetable sources; for this reason, it has been widely studied. However, few studies have compared the potency of individual ACNs (111, 115, 131, 132), raising the question of whether compounds other than cyanidin could also be helpful for specific biological activities. From our search for the last 5 years of studies on single ACNs, seven of the entries tried a cyanidin-based compound, seven a delphinidin-based compound, three a pelargonidin-based compound, two a malvidin-based compound, and none of them a peonidin-based compound. Structure–activity relationships have explored the influence of glycosylation on the biological activity of ACNs and compared their antioxidant activity (133, 134). However, further explorations and comparisons for individual compounds linking anti-inflammatory and obesity-related biological activities are needed to better understand their relative efficacies.

Various studies have addressed whether ACNs have specific molecular targets regarding chemical structure. For instance, through docking-based virtual screening, Liu et al. (135) found that cyanidin has a binding site in the IL-17A receptor subunit (IL-17RA). Cyanidin binding to this site inhibits IL-17A-induced gene expression in human and mouse cells by inhibiting the IL-17RA interaction with the IL-17A interleukin. Furthermore, they showed that cyanidin inhibits IL-17A-dependent skin hyperplasia and attenuates airway inflammation in mouse models of steroid-resistant and severe asthma. Besides IL-17RA, cyanidin-3-*O*-glucoside was also shown to interact with ligand binding domains of PPARα, -γ, and δ/β by surface plasmon resonance (115). In this study, pelargonidin-3-*O*-glucoside and delphinidin-3-*O*-glucoside were also shown to interact with PPAR ligand binding domains. Cyanidin-3-*O*-arabinoside, cyanidin-3-*O*-galactoside, peonidin-3-*O*-arabinoside, and peonidin-3-*O*-galactoside were also shown to interact with and inhibit pancreatic lipase (132); from them, cyanidin-3-*O*-arabinoside was shown to impact significantly secondary structures of the enzyme. Finally, the generic ACN core structure without substituents was shown to interact with 3-hydroxy-3-methylglutaryl coenzyme A (HMG-CoA) reductase and acyl-CoA cholesterol acyltransferase (ACAT) proteins through molecular docking (136).

**Figure 5:**
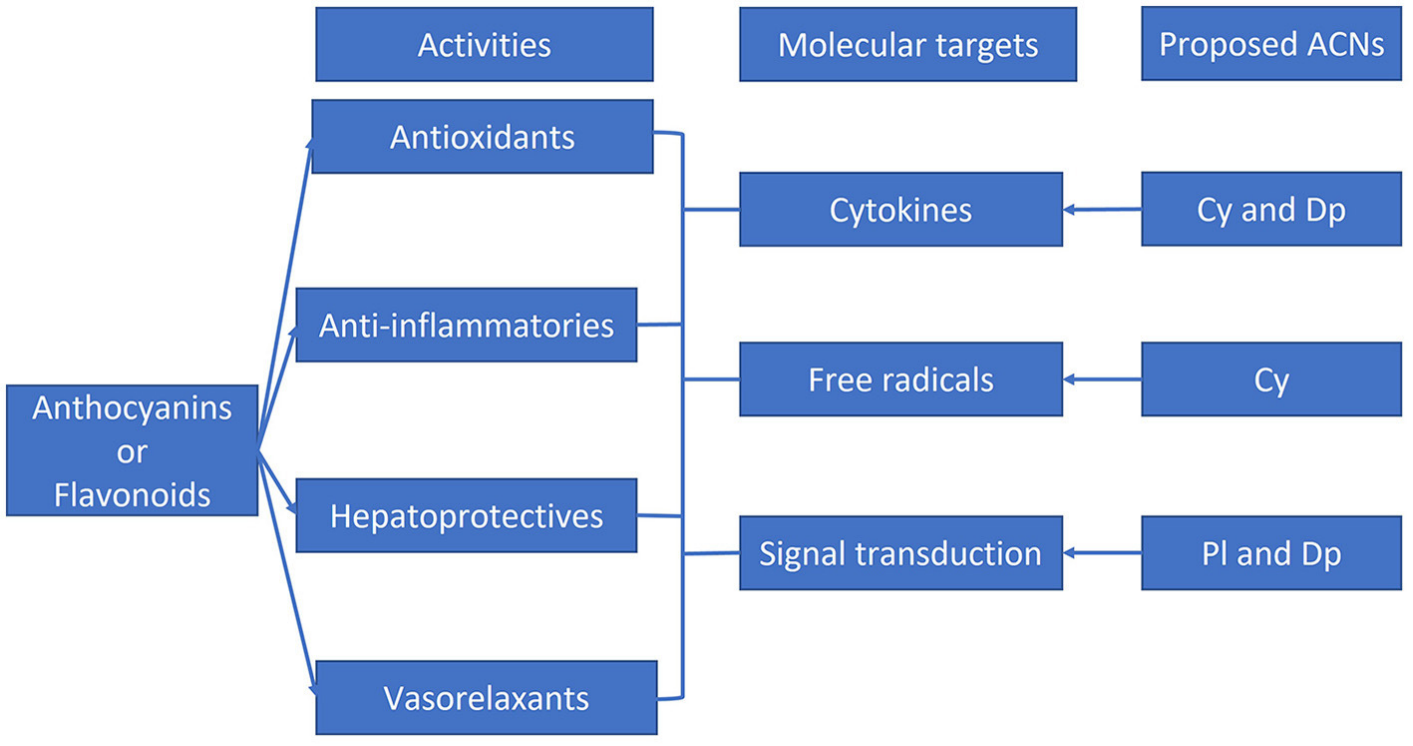
Graphic depiction of some beneficial effects and molecular targets reported for flavonoids and anthocyanins. Single anthocyanins likely correlated with specific molecular targets are shown. Adapted from Azzini et al. (2), Gomes et al. (3), Abou Baker (4), Fallah et al. (5), and Mozaffarian and Wu (6).

From all the molecular targets and pathways discussed in this review, selected more representative associations with single ACNs are shown in Figure 5. Further studies on single ACNs or their metabolites should corroborate or modify these suggestions.

## 6. Perspectives and conclusion

Knowing the ACN profile composition of matrices used in CT provides valuable information to partially elucidate individual ACN mechanisms of action. This information would allow us to design food formulations rich in effective bioactive compounds or to select species and cultivars with optimal composition for specific diseases and bioactivities. The ACN bioavailability was reported to be higher for a unique dose than for the same dose divided throughout the day. However, more marked effects were observed in CTs that used low doses over long periods and in cohorts with higher baselines for most of the studied markers, coinciding with advanced pathological features. From our bioavailability and principal components analyses, we found that delphinidin has been shown to reach the highest plasma concentrations and consistently have a significant dose-dependent correlation with HDL levels. From our analysis of pure single anthocyanins studies, pelargonidin showed promising potency and molecular target identification results.

Therefore, we propose that the next steps in ACN research should focus on standardizing doses, identifying individual compounds, and developing delivery strategies to cope with ACN degradation. To better understand mechanisms of action ruling ACN bioactivities, investigating ACN-derived metabolites is also a promising research area.

## Author contributions

JF-M: Conceptualization, Data curation, Investigation, Methodology, Visualization, Writing—original draft. SL: Formal analysis, Investigation, Methodology, Writing—review & editing. AD-U: Investigation, Writing—original draft. DL-V: Conceptualization, Funding acquisition, Project administration, Supervision, Writing—review & editing. NG: Conceptualization, Funding acquisition, Supervision, Writing—review & editing.
