# Supplementary material for: Single anthocyanins effectiveness modulating inflammation markers in obesity: dosage and matrix composition analysis
Source: Front Nutr. 2023 Nov 2;10:1255518. doi: 10.3389/fnut.2023.1255518 (PMC10651755; doi:10.3389/fnut.2023.1255518)
Supplement: Supplementary file 1 [file Data_Sheet_1.docx]

*SUPPLEMENTARY FIGURES AND TABLES FOR*

**Single anthocyanins effectiveness modulating inflammation markers in obesity: dosage and matrix composition analysis**

**Jorge Alberto Fragoso-Medina, Selma Romina López Vaquera, Astrid Domínguez-Uscanga, Diego Armando Luna-Vital and Noemí García**

*Supplementary figure S1: Partial correlations between Ac(a), Cy(b), Dp(c), Mv(d), Pn(e), and Pt(f) doses levels and most commonly measured markers in the included clinical studies in Figure 2. Pearson's correlation coefficients were estimated; a p-value < 0.05 was considered significant (*). Corresponding statistical estimations can be found in Supplementary Table 2 (ST2). Ac, acylated anthocyanin; Cy, cyanidin; DBP, diastolic blood pressure; Dp, delphinidin; Glu, glucose; HDL, high-density lipoprotein; LDL, low-density lipoprotein; Mv, malvidin; Pn, peonidin; Pt, petunidin; SBP, systolic blood pressure; TG, triglycerides.*

*Supplementary table ST1: Abbreviations used in figure 2.*

*Supplementary table ST2: Statistical estimations for Figure S1. Ac, acylated anthocyanin; Cy, cyanidin; DBP, diastolic blood pressure; Dp, delphinidin; Glu, glucose; HDL, high-density lipoprotein; LDL, low-density lipoprotein; Mv, malvidin; Pn, peonidin; Pt, petunidin; SBP, systolic blood pressure; TG, triglycerides.*

*Supplementary figure S1*

*
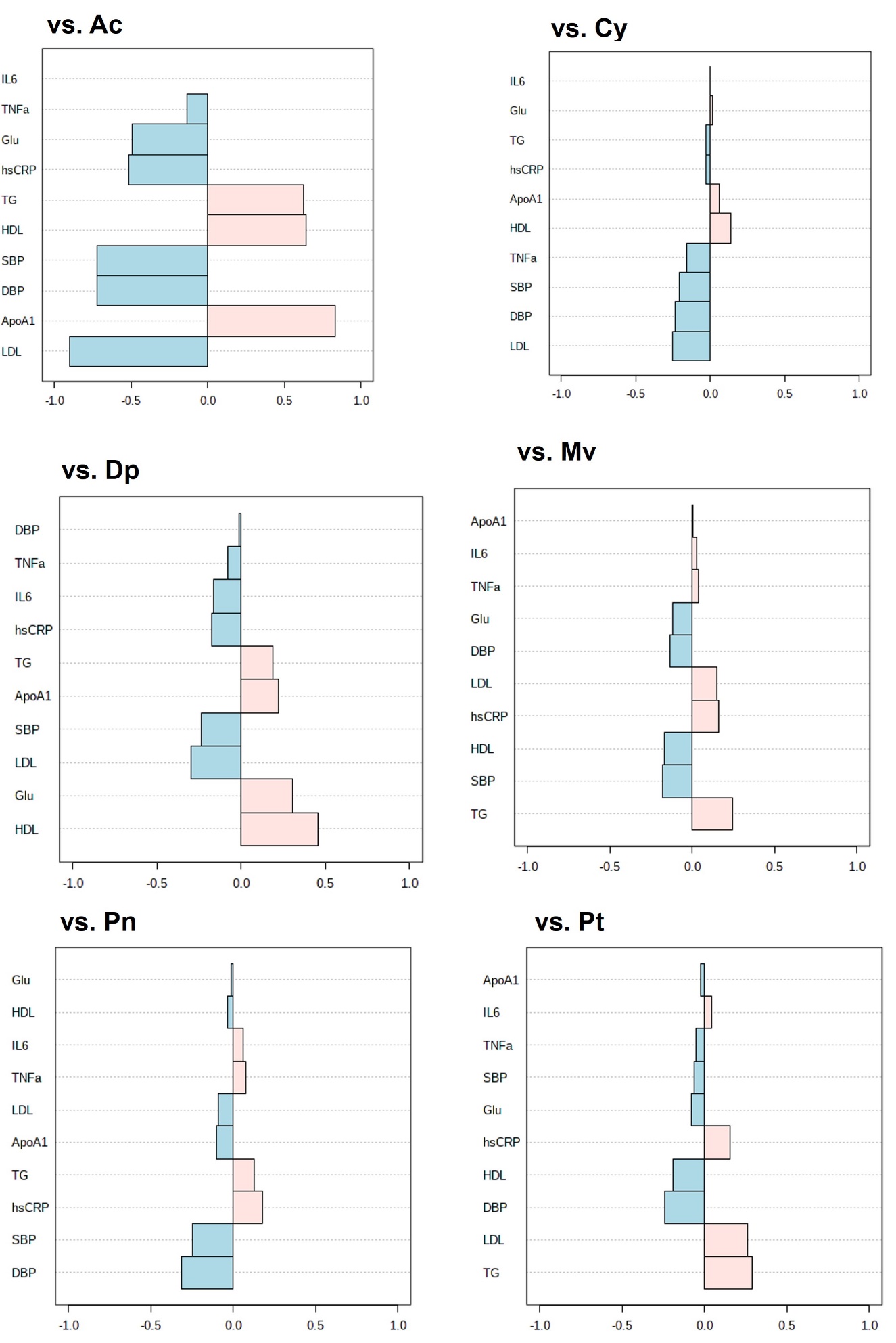
*

*Supplementary table ST1*

| *Ac* | *Acylated anthocyanins* |
| --- | --- |
| *Cy* | *Cyanidin* |
| *Dp* | *Delphinidin* |
| *Mv* | *Malvidin* |
| *Pl* | *Pelargonidin* |
| *Pn* | *Peonidin* |
| *Pt* | *Petunidin* |
| *Proant* | *Proanthocyanidins* |
| *hsCRP* | *High sensitive C-Reactive Protein* |
| *DBP* | *Diastolic Blood Pressure* |
| *Ins_AUC* | *Area under curve of plasma insulin* |
| *Apo-A1* | *Apolipoprotein A1* |
| *Chol_eflux* | *Cholesterol efflux to serum from macrophages* |
| *SBP* | *Systolic Blood Pressure* |
| *Apo_ B48* | *Apolipoprotein B48* |
| *HOMA_IR* | *Homeostatic Model Assessment-Insulin Resistance* |
| *MCP_1* | *Macrophages Chemoatractant Protein 1* |
| *TG* | *Triacyl glycerides* |
| *LDL* | *Low Density Lipoprotein* |
| *Chol_HDL* | *Cholesterol/ High Density Lipoprotein Ratio* |
| *HDL* | *High Density Lipoprotein* |
| *P_sel* | *P-Selectin* |
| *Glu* | *Glucose* |
| *Ins* | *Insulin* |
| *HbA!c_Glu* | *Glycosylated Hemoglobin A1c* |
| *ox_LDL* | *Oxidized Low Density Lipoprotein* |
| *IL10* | *Interleukin 10* |
| *MDA_HNE* | *Malondialdehyde and/or 4-Hydroxynonenal levels* |
| *IL8* | *Interleukin 8* |
| *VCAM1* | *Vascular Cell Adhesion Molecule 1* |
| *TNFα* | *Tumor Necrosis Factor α* |
| *sTNFα_R* | *Soluble Tumor Necrosis Factor α Receptor* |
| *IL1B* | *Interleukin 1B* |
| *IL6* | *Interleukin 6* |

*Supplementary table ST2*

| Compound | Marker | correlation (Pearson) | t-stat | p-value | FDR |
| --- | --- | --- | --- | --- | --- |
| Ac | LDL | -0.25414 | -1.5322 | 0.13473 | 0.74334 |
| Ac | DBP | -0.23551 | -1.413 | 0.16675 | 0.74334 |
| Ac | SBP | -0.20821 | -1.2413 | 0.223 | 0.74334 |
| Ac | TNFa | -0.15581 | -0.91973 | 0.36419 | 0.85135 |
| Ac | HDL | 0.13698 | 0.80629 | 0.42568 | 0.85135 |
| Ac | ApoA1 | 0.061006 | 0.35638 | 0.72375 | 0.98412 |
| Ac | hsCRP | -0.029687 | -0.17318 | 0.86354 | 0.98412 |
| Ac | TG | -0.027136 | -0.15828 | 0.87517 | 0.98412 |
| Ac | Glu | 0.016759 | 0.097734 | 0.92272 | 0.98412 |
| Ac | IL6 | -0.0034378 | 0.020046 | 0.98412 | 0.98412 |
| Cy | LDL | -0.25414 | -1.5322 | 0.13473 | 0.74334 |
| Cy | DBP | -0.23551 | -1.413 | 0.16675 | 0.74334 |
| Cy | SBP | -0.20821 | -1.2413 | 0.223 | 0.74334 |
| Cy | TNFa | -0.15581 | -0.91973 | 0.36419 | 0.85135 |
| Cy | HDL | 0.13698 | 0.80629 | 0.42568 | 0.85135 |
| Cy | ApoA1 | 0.061006 | 0.35638 | 0.72375 | 0.98412 |
| Cy | hsCRP | -0.029687 | -0.17318 | 0.86354 | 0.98412 |
| Cy | TG | -0.027136 | -0.15828 | 0.87517 | 0.98412 |
| Cy | Glu | 0.016759 | 0.097734 | 0.92272 | 0.98412 |
| Cy | IL6 | -0.0034378 | 0.020046 | 0.98412 | 0.98412 |
| Dp | HDL | 0.45618 | 2.6139 | 0.014692 | 0.14692 |
| Dp | Glu | 0.30719 | 1.6459 | 0.11181 | 0.41599 |
| Dp | LDL | -0.29703 | -1.5861 | 0.1248 | 0.41599 |
| Dp | SBP | -0.23442 | -1.2295 | 0.22988 | 0.49989 |
| Dp | ApoA1 | 0.22209 | 1.1614 | 0.25602 | 0.49989 |
| Dp | TG | 0.18918 | 0.98235 | 0.33498 | 0.49989 |
| Dp | hsCRP | -0.17312 | -0.89628 | 0.37833 | 0.49989 |
| Dp | IL6 | -0.16553 | -0.85583 | 0.39991 | 0.49989 |
| Dp | TNFa | -0.077007 | -0.39383 | 0.69692 | 0.77435 |
| Dp | DBP | -0.014926 | 0.076117 | 0.93991 | 0.93991 |
| Mv | TG | 0.24342 | 1.2295 | 0.23081 | 0.803 |
| Mv | SBP | -0.17765 | -0.88436 | 0.38527 | 0.803 |
| Mv | HDL | -0.16684 | -0.82895 | 0.4153 | 0.803 |
| Mv | hsCRP | 0.1584 | 0.78591 | 0.43961 | 0.803 |
| Mv | LDL | 0.14982 | 0.74235 | 0.46508 | 0.803 |
| Mv | DBP | -0.13476 | -0.66625 | 0.5116 | 0.803 |
| Mv | Glu | -0.11915 | -0.58789 | 0.5621 | 0.803 |
| Mv | TNFa | 0.039465 | 0.19349 | 0.84821 | 0.97681 |
| Mv | IL6 | 0.026845 | 0.13156 | 0.89643 | 0.97681 |
| Mv | ApoA1 | 0.0059952 | 0.029371 | 0.97681 | 0.97681 |
| Pn | DBP | -0.3167 | -1.566 | 0.13161 | 0.95301 |
| Pn | SBP | -0.24535 | -1.1871 | 0.24786 | 0.95301 |
| Pn | hsCRP | 0.17583 | 0.83776 | 0.41118 | 0.95301 |
| Pn | TG | 0.1288 | 0.60918 | 0.54864 | 0.95301 |
| Pn | ApoA1 | -0.10284 | -0.48495 | 0.63251 | 0.95301 |
| Pn | LDL | -0.091599 | -0.43145 | 0.67034 | 0.95301 |
| *Supplementary table ST2 (continuation)* | | | | | |
| Pn | TNFa | 0.078632 | 0.36996 | 0.71495 | 0.95301 |
| Pn | IL6 | 0.061109 | 0.28716 | 0.77667 | 0.95301 |
| Pn | HDL | -0.036039 | -0.16915 | 0.86723 | 0.95301 |
| Pn | Glu | -0.012707 | 0.059604 | 0.95301 | 0.95301 |
| Pt | TG | 0.29125 | 1.5222 | 0.1405 | 0.75734 |
| Pt | LDL | 0.26287 | 1.3623 | 0.18526 | 0.75734 |
| Pt | DBP | -0.24035 | -1.238 | 0.2272 | 0.75734 |
| Pt | HDL | -0.18956 | -0.96532 | 0.34363 | 0.85908 |
| Pt | hsCRP | 0.15699 | 0.79481 | 0.4342 | 0.86841 |
| Pt | Glu | -0.079337 | -0.39794 | 0.69405 | 0.90696 |
| Pt | SBP | -0.064023 | -0.32077 | 0.75105 | 0.90696 |
| Pt | TNFa | -0.052715 | -0.26394 | 0.79399 | 0.90696 |
| Pt | IL6 | 0.042187 | 0.21112 | 0.8345 | 0.90696 |
| Pt | ApoA1 | -0.023605 | -0.11806 | 0.90696 | 0.90696 |
